# Acetylcholine Reduces I_Kr_ and Prolongs Action Potentials in Human Ventricular Cardiomyocytes

**DOI:** 10.3390/biomedicines10020244

**Published:** 2022-01-24

**Authors:** István Koncz, Arie O. Verkerk, Michele Nicastro, Ronald Wilders, Tamás Árpádffy-Lovas, Tibor Magyar, Noémi Tóth, Norbert Nagy, Micah Madrid, Zexu Lin, Igor R. Efimov

**Affiliations:** 1 Department of Biomedical Engineering, The George Washington University, Washington, DC 20052, USA; koncz.istvan@med.u-szeged.hu (I.K.); mmadrid@gwu.edu (M.M.); zexulin@gwu.edu (Z.L.); 2 Department of Pharmacology and Pharmacotherapy, Albert Szent-Györgyi Medical School, University of Szeged, 6721 Szeged, Hungary; arpadffy-lovas.tamas@med.u-szeged.hu (T.Á.-L.); magyti@gmail.com (T.M.); toth.noemi@med.u-szeged.hu (N.T.); nagy.norbert@med.u-szeged.hu (N.N.); 3 Heart Center, Department of Experimental Cardiology, Amsterdam Cardiovascular Sciences, Amsterdam UMC, University of Amsterdam, 1105 AZ Amsterdam, The Netherlands; a.o.verkerk@amsterdamumc.nl (A.O.V.); m.nicastro@amsterdamumc.nl (M.N.); 4 Department of Medical Biology, Amsterdam Cardiovascular Sciences, Amsterdam UMC, University of Amsterdam, 1105 AZ Amsterdam, The Netherlands; r.wilders@amsterdamumc.nl; 5 ELKH-SZTE Research Group of Cardiovascular Pharmacology, 6721 Szeged, Hungary; 6 Querrey Simpson Institute for Bioelectronics, Northwestern University, Chicago, IL 60611, USA

**Keywords:** acetylcholine, action potential duration, delayed rectifier K^+^ current (I_Kr_), human induced pluripotent stem-cell-derived cardiomyocytes (hiPSC-CMs), repolarization

## Abstract

Vagal nerve stimulation (VNS) has a meaningful basis as a potentially effective treatment for heart failure with reduced ejection fraction. There is an ongoing VNS randomized study, and four studies are completed. However, relatively little is known about the effect of acetylcholine (ACh) on repolarization in human ventricular cardiomyocytes, as well as the effect of ACh on the rapid component of the delayed rectifier K^+^ current (I_Kr_). Here, we investigated the effect of ACh on the action potential parameters in human ventricular preparations and on I_Kr_ in human induced pluripotent stem-cell-derived cardiomyocytes (hiPSC-CMs). Using standard microelectrode technique, we demonstrated that ACh (5 µM) significantly increased the action potential duration in human left ventricular myocardial slices. ACh (5 µM) also prolonged repolarization in a human Purkinje fiber and a papillary muscle. Optical mapping revealed that ACh increased the action potential duration in human left ventricular myocardial slices and that the effect was dose-dependent. Perforated patch clamp experiments demonstrated action potential prolongation and a significant decrease in I_Kr_ by ACh (5 µM) in hiPSC-CMs. Computer simulations of the electrical activity of a human ventricular cardiomyocyte showed an increase in action potential duration upon implementation of the experimentally observed ACh-induced changes in the fully activated conductance and steady-state activation of I_Kr_. Our findings support the hypothesis that ACh can influence the repolarization in human ventricular cardiomyocytes by at least changes in I_Kr_.

## 1. Introduction

Heart failure with a reduced ejection fraction is a leading cause of morbidity and mortality and exceeds 2% of the general population [1]. Significant healthcare improvements have been made for heart failure patients [2], but mortality risk remains high, and many patients remain very symptomatic [3]. Heart failure patients have a major disturbance of autonomic function, including a reduced parasympathetic control, and pharmacological augmentation of parasympathetic tone in patients with heart failure was suggested [4]. Direct stimulation of the vagus nerve to improve parasympathetic tone is also possible [5], and in the last decade, series of clinical trials have been designed and carried out, and one is currently underway, to evaluate the effect of vagal nerve stimulation (VNS) on heart failure [6,7]. Increased parasympathetic tone results in increase in acetylcholine (ACh) levels [8]. The effects of ACh on the electrophysiology and contractility of supraventricular human tissue are well-known, but in ventricular tissue it is not completely understood [9,10].

In human atrial preparations and isolated human atrial cardiomyocytes, ACh results in a negative inotropic effect and action potential (AP) shortening due to a well-known activation of the ACh-sensitive K^+^ current (I_K,ACh_) and reduction in the L-type Ca^2+^ current (I_Ca,L_) [11,12]. The ACh effects on human ventricular tissue and cardiomyocytes are less clear. Some papers report no effects of ACh on APs and contractions of ventricular papillary muscle from men [13], while others report reduced contractions in response to the muscarinic receptor agonist, carbachol [14,15]. In some studies, performed on trabeculae obtained from non-diseased human hearts, ACh (10^−9^ to 10^−4^ M; 1 nM to 100 µM) even elicited a positive inotropic effect on the baseline ventricular force of contraction [16,17]. It is also reported that intracoronary administration of ACh to a patient who had normal QT interval, which corresponds to ventricular AP duration (APD), unmasked abnormal QT interval prolongation and induced torsades de pointes (TdP) [18]. In addition, the same research group found that intracoronary administration of ACh induced prolongation of monophasic AP (MAP) duration and caused TdP in a patient in whom intravenous atropine administration did not induce any change in MAP duration [19]. 

AP prolongation in response to ACh administration was also found in some animal studies. Litovsky and Antzelevitch found that ACh (0.1 and 1 µM) significantly increased the canine subepicardial, but not the subendocardial, APD measured at 90% repolarization (APD_90_) at a basic cycle length (BCL) of 500 ms [20]. In addition, Gilmour and Zipes demonstrated the ability of ACh (10 µM) to increase APD measured at 50% repolarization (APD_50_) and tension in dog Purkinje fibers [21]. Finally, carbachol and ACh were found to increase the APDs in rabbit sinoatrial node (SAN) cells [22,23]. Interestingly, the tail amplitude of delayed rectifier K^+^ currents was decreased by carbachol in SAN cells [23]. Similar effects were found in guinea-pig papillary muscle [24] and guinea-pig ventricular myocytes during isoproterenol stimulation [25]. A study using guinea-pig SAN cells demonstrated that at least the slow component (I_Ks_) contributes to the muscarinic receptor stimulation induced decrease in delayed rectifier K^+^ currents [26]. Studies of the effects of ACh on the rapid component of the delayed K^+^ rectifier (I_Kr_) are yet to be performed in cardiomyocytes. 

In the present study, we investigated the effects of ACh on AP parameters registered in human ventricular preparations and in ventricular cardiomyocytes derived from human induced pluripotent stem cells (hiPSC-CMs). In addition, we studied the effects of ACh on I_Kr_ in hiPSC-CMs. We found that ACh significantly increased the APD in human left ventricular myocardial slices and hiPSC-CMs. We also found that ACh significantly decreased the I_Kr_ in hiPSC-CMs and that the APD of the O’Hara–Rudy model human ventricular cardiomyocyte [27] increased upon implementation of the experimentally observed ACh-induced changes in conductance and kinetics of I_Kr_.

## 2. Materials and Methods

### 2.1. Human Heart Measurements

#### 2.1.1. Standard Microelectrode Technique

##### 2.1.1.1. Human Papillary Muscle and Purkinje Fiber

A human heart that was unsuitable for transplantation (a donor heart with 29% ejection fraction) was obtained from an organ donor. The investigation conformed to the principles outlined in the Declaration of Helsinki of the World Medical Association. All experimental protocols were approved by the Scientific and Research Ethical Committee of the Medical Scientific Board at the Hungarian Ministry of Health (ETT-TUKEB), under ethical approval No. 4991-0/2010-1018EKU (339/PI/010). Experiments of deidentified unsuitable donor hearts were also approved by the Institutional Review Board of the George Washington University (Washington, DC, USA) and Washington Regional Transplant Community (Falls Church, VA, USA).

Human cardiac tissue was stored at 4 °C in cardioplegic solution containing (in mM): NaCl 110, KCl 16, MgCl_2_ 16, NaHCO_3_ 10, CaCl_2_ 1.2. Ventricular (papillary) muscle and Purkinje fiber were obtained from right ventricle. The Purkinje fiber and the papillary muscle were placed in Locke’s solution containing (in mM): NaCl 120, KCl 4, CaCl_2_ 2, MgCl_2_ 1, NaHCO_3_ 22, glucose 11. The tissue was allowed to equilibrate for at least 2 h during continuous superfusion (flow rate 4–5 mL/min) with Locke’s solution. The solution was gassed with 95% O_2_ and 5% CO_2_ at 37 °C. At impalement, the preparations were observed under a surgical microscope (Purkinje fiber was examined by Zeiss OPMI PRO). The Purkinje fiber was stimulated at a basic cycle length (BCL) of 500 ms and the papillary muscle at a BCL of 1000 ms, and both were allowed to equilibrate for at least 2 h while they were continuously superfused with Locke’s solution. Electrical pulses of 2 ms in duration and twice diastolic threshold in intensity were delivered to the preparations through bipolar platinum electrodes. Transmembrane potentials were recorded with the use of glass capillary microelectrodes filled with 3 M KCl (tip resistance: 5 to 15 MΩ). The microelectrodes were coupled through an Ag–AgCl junction to the input of a high-impedance, capacitance-neutralizing amplifier (Experimetria Ltd., Budapest, Hungary). Intracellular recordings were displayed on a storage oscilloscope (Hitachi V-555) and led to a computer system (APES) designed for on-line determination of the following parameters: resting membrane potential (RMP), AP amplitude (APA), APD at 10, 25, 50, 75, and 90% repolarization (APD_10_, APD_25_, APD_50_, APD_75_, and APD_90_, respectively), and the maximum rate of rise of the AP upstroke (V_max_). Control recordings were obtained after an equilibrium period. The effects of ACh were determined at 5 µM. For all experiments ACh was purchased from Sigma/Merck.

##### 2.1.1.2. Human Left Ventricular Myocardial Slices

Left ventricular tissue slices as used in the experiments of Section 3.1.1 were prepared from 3 non-pathological human hearts as described in Section 2.1.2 below. AP recordings were made as described in Section 2.1.1.1 above, but with the use of a Zeiss Stemi SV 11 microscope, a Power 1401 data acquisition interface and Spike 2 software (Cambridge Electronic Design Ltd., Cambridge, UK), and an Electro 705 intracellular amplifier (WPI). The preparations were superfused with oxygenated (95% O_2_ and 5% CO_2_ at 37 ± 0.5 °C) Tyrode’s solution containing (in mM): NaCl 129, KCl 4.7, NaH_2_PO_4_ 1.19, NaHCO_3_ 20, CaCl_2_ 1.3, MgCl_2_ 1.05, glucose 11.1; pH 7.4.

The left ventricular tissue slice as used in the experiment of Section 3.4.2 was prepared from another non-pathological human heart as follows. A piece from the basal part of the left ventricle was glued with tissue adhesive directly to top of the cutting stage of a vibratome (Vibratome 3000 PELCO 100 Vibratome Sectioning System, generous donation from Mr. Tamás Leisztinger). A tangential slice (400 µm) was cut in cold (4 °C) Locke’s solution with a steel blade. The slice was placed in a preincubation chamber filled with oxygenated Locke’s solution at room temperature for at least 3 h. AP recordings were made as described in Section 2.1.1.1 above. Dofetilide was purchased from Carbosynth Ltd., Compton, UK.

#### 2.1.2. Human Myocardial Slices and Optical Imaging

Human donor hearts unsuitable for transplantation were obtained from the Washington Regional Transplant Community (WRTC). The preparation of left ventricular (LV) slices has been described previously [28]. Briefly, LV tissue was isolated, and approximately 1 cm^3^ cubes were cut in 4 °C cardioplegic solution and attached to the tissue holder of a vibrating microtome (7000 smz-2, Campden Instruments Ltd., Loughborough, UK). LV slices (400 μm thickness) were sectioned at 80 Hz frequency and 0.04 mm/s speed in 4 °C slicing solution containing (in mM): NaCl 140, KCl 6, MgCl_2_ 1, CaCl_2_ 1.8, glucose 10, HEPES 10; pH 7.4. 2,3-Butanedione (10 mM) was added to suppress contraction. Slices were then incubated for at least 20 min at room temperature in recovering solution (containing (in mM): NaCl 140, KCl 4.5, MgCl_2_ 1, CaCl_2_ 1.8, glucose 10, HEPES 10, BDM 10; pH 7.4) and then used in acute studies. To optically map LV slices, slices were pinned down (Minutien Pins, 26002-10, Fine Science Tools, Foster City, CA, USA) to a bath containing Tyrode’s solution at 37 °C and paced using a homemade bipolar platinum electrode (Coated Platinum-Iridium Wire, 778000, A-M Systems, Sequim, WA, USA) at 1.5× voltage threshold of stimulation, 2 ms pulse width and frequencies ranging from 0.5 Hz to loss of 1:1 capture. Blebbistatin (10–15 mM) was added to Tyrode’s solution during optical mapping to arrest motion. Slices were incubated in RH237 (voltage sensitive dye, Biotium, Fremont, CA, USA, 61018) and Rhod2-AM (calcium indicator dye, Thermo Fisher Scientific, Waltham, MA, USA, R1244) sequentially and excited with a green LED light source (520 ± 17 nm). Emitted light was collected using a tandem lens optical mapping system and recorded at 1000 Hz using two CMOS cameras (Ultima-L, SciMedia, Costa Mesa, CA, USA). Data analysis was performed using a custom MATLAB program.

### 2.2. Cellular Electrophysiology in hiPSC-CMs

#### 2.2.1. Preparation of hiPSC-CMs for Electrophysiology

I_Kr_ and single cell APs were measured in hiPSC-CMs, which are becoming a well-established human cell source for cardiac disease modeling [29,30] and drug screening [31]. hiPSC-CMs were generated from the control hiPSC line LUMC0099iCTRL04, which was derived from skin fibroblasts of a Caucasian woman [32], using an mRNA based reprogramming method [33]. The LUMC0099iCTRL04 line is registered in the Human Pluripotent Stem Cell Registry (hPSCreg), which contains all details pertaining to its generation and characterization [34]. Differentiation to CMs was performed in B27-enriched RPMI medium following a protocol based on small-molecule-mediated canonical Wnt pathway modulation with CHIR99021 and IWP4, as we reported previously [35]. The differentiating hiPSC cultures were enriched for cardiomyocytes through metabolic selection by adding lactic acid in substitution of glucose [35]. hiPSC-CM cultures were dissociated into single cells at day 21 using an enzymatic treatment with trypsin 0.05% for 25 min whereafter trypsin was inactivated by serum enriched medium, i.e., B27-enriched RPMI with the addition of 10% fetal bovine serum (FBS). Subsequently, the single cells were cryopreserved as described previously [36] in B27-enriched RPMI medium with the addition of 50% FBS and 10% DMSO. One week before the patch clamp experiments were performed, a vial with frozen cells was incubated at 37 °C, and the thawed cells were slowly transferred to a conical tube containing 9 mL of their medium with the addition of 10% FBS and resuspended. Cells were precipitated at 250 g for 3 min and resuspended in B27-enriched RPMI containing 10% FBS, 1% pen/strep and ROCK inhibitor. The thawed hiPSC-CMs were plated at a low density (≈5 × 10^4^ cells) on Matrigel-coated glass coverslips, in B27-enriched RPMI containing 10% FBS, 1% pen/strep and ROCK inhibitor. Approximately 24 h after plating the medium was changed into B27-enriched RPMI containing 1% pen/strep, which was refreshed every 2–3 days until the experiment was performed. Spontaneously beating hiPSC-CMs showing regular, synchronous contractions were selected for patch clamp recordings.

#### 2.2.2. Data Acquisition

I_Kr_ and APs were recorded at 36 ± 0.2 °C with the perforated patch clamp method using an Axopatch 200 B amplifier (Molecular Devices, Sunnyvale, CA, USA). Voltage control, data acquisition, and analysis were realized with custom software. Extracellular solution contained (in mM): NaCl 140, KCl 5.4, CaCl_2_ 1.8, MgCl_2_ 1.0, glucose 5.5, HEPES 5.0; pH 7.4 (NaOH). Pipettes (borosilicate glass, 2–3 MΩ; Harvard Apparatus, Waterbeach, UK) were filled with solution containing (in mM): K-gluconate 125, KCl 20, NaCl 5, amphotericin-B 0.44, HEPES 10; pH 7.2 (KOH). Potentials were corrected for the calculated liquid junction potential [37]. Cell membrane capacitance (C_m_) was calculated by dividing the time constant of the decay of the capacitive transient after a −5 mV voltage step from −40 mV by the series resistance. I_Kr_ signals were lowpass-filtered with a cutoff of 5 kHz and digitized at 5 kHz; APs were filtered and digitized at 5 and 40 kHz, respectively. Series resistance was compensated for by at least 80%. 

#### 2.2.3. Delayed Rectifier K^+^ Current Measurements 

I_Kr_ was measured using 4 s long depolarizing pulses from a holding potential of −50 mV. Cycle length was 10 s. JNJ303 (0.5 mM) and 5 µM nifedipine were present to block the slow component of the delayed rectifier K^+^ current (I_Ks_) and the L-type Ca^2+^ current (I_Ca,L_), respectively. Currents were recorded under baseline conditions, in the presence of 5 µM ACh, and in the presence of ACh and 5 µM E4031, to block I_Kr_ completely. I_Kr_ was defined as the current sensitive to 5 µM E4031 and was analyzed at the end of the depolarizing pulses and as maximal tail current induced by stepping back to the −50 mV holding potential. Currents in current–voltage (I-V) relationships were normalized to the largest current amplitudes under baseline conditions during depolarization pulses and tails, respectively. Current density was calculated by dividing the current amplitude by C_m_. Steady-state activation curves were fitted using the Boltzmann equation:

I/I_max_ = A/{1.0 + exp[(V_1/2_ − V)/k]}, (1)

to determine V_1/2_ (membrane potential for the half-maximal activation) and the slope factor k (in mV). The time course of deactivation was fitted by a double-exponential equation:

I/I_max_ = A_f_ × exp(−t/τ_f_) + A_s_ × exp(−t/τ_s_), (2)

where A_f_ and A_s_ are the fractions of the fast and slow deactivation components, and τ_f_ and τ_s_ are the time constants of the fast and slow deactivating components, respectively. The time course of activation was fitted by the mono-exponential equation:

I/I_max_ = A × [1 − exp(−t/τ)], (3)

where A and τ are the amplitude and time constant of the activating current.

#### 2.2.4. Action Potential Measurements 

APs were measured with dynamic clamp [38] to inject an in silico I_K1_ with an I-V relationship of Kir2.1 channels with a 2 pA/pF outward peak [35], resulting in quiescent hiPSC-CMs with a resting membrane potential (RMP) of −80 mV or more negative. JNJ303 (0.5 mM) was present to block I_Ks_. APs were elicited at 1 and 3 Hz by 3 ms, ≈1.3× threshold current pulses through the patch pipette. The RMP; maximal AP upstroke rate (V_max_); AP amplitude (APA); AP duration at 20, 50, and 90% repolarization (APD_20_, APD_50_, APD_90_); and maximal phase-3 repolarization rate were analyzed. V_max_ and maximal phase-3 repolarization rate were analyzed from the first derivative of the AP upstroke and phase-3 repolarization, respectively, and are expressed in V/s. Parameters from 10 consecutive APs were averaged.

### 2.3. Computer Simulations

Functional effects of the ACh-induced reduction in I_Kr_ on human ventricular cardiomyocytes were assessed by computer simulations using the O’Hara–Rudy human ventricular cell model [27]. The effects of 5 µM ACh on I_Kr_ were incorporated by a 23% decrease in its fully activated conductance and a +17.3 mV shift in its steady-state activation curve. The CellML code of the O’Hara–Rudy model, as available from the CellML Model Repository [39] at https://www.cellml.org/ (accessed on 29 October 2021), was edited and run in version 0.9.31.1409 of the Windows-based Cellular Open Resource (COR) environment [40]. All simulations were run for a period of 110 s, which appeared a sufficiently long time to reach steady-state behavior. The analyzed data are from the final 10 s of the 110-s period. 

### 2.4. Statistics

Data are expressed as mean ±SEM. Statistical analysis was carried out with SigmaStat 3.5 software (Systat Software, Inc., San Jose, CA, USA). Normality and equal variance assumptions were tested with the Kolmogorov–Smirnov and the Levene median test, respectively. The paired *t*-test (for normally distributed data) and the Wilcoxon signed rank test (for non-normally distributed data) were used for two group comparisons. One-way repeated measures ANOVA followed by pairwise comparison using the Student–Newman–Keuls test was used to compare action potential parameters at different time points of exposure to ACh. Two-way repeated measures ANOVA followed by pairwise comparison using the Student–Newman–Keuls test was used for group comparisons in the I-V relationship curves. *p* < 0.05 was considered statistically significant.

## 3. Results

### 3.1. Standard Microelectrode Measurements

#### 3.1.1. Human Myocardial Slice Preparations

**Figure 1:**
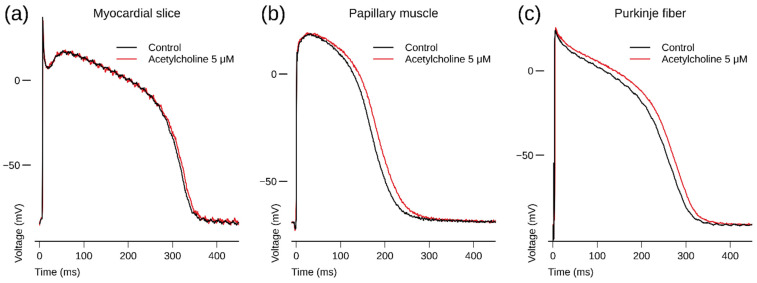
(**a**) Human left ventricular myocardial slice: superimposed recording of action potentials (APs) during control and after 3 min superfusion with ACh (5 µM) at a BCL of 1000 ms; (**b**) human right ventricular papillary muscle: superimposed recording of APs during control and after 3 min exposure to ACh (5 µM) at a BCL of 1000 ms; (**c**) human right ventricular Purkinje fiber: superimposed recording of an AP during control and after 3 min exposure to ACh (5 µM) at a BCL of 500 ms. Note that ACh slightly prolongs repolarization. AP waveform recordings originate from the same preparation and same impalement. Panel a depicts APs recorded from a non-diseased human donor heart; panels b and c depict APs recorded from a human donor heart with 29% ejection fraction.

In human myocardial slice preparations taken from three donor hearts, ACh (5 µM) slightly but statistically significantly increased APD at 75 and 90% repolarization (APD_75_ and APD_90_) after 3 and 5 min exposure (Table 1, Figure 1a).

#### 3.1.2. Human Purkinje Fiber

In a human Purkinje fiber (taken from a donor heart with 29% ejection fraction), ACh increased APD after 3 min exposure (Figure 1c). 

#### 3.1.3. Human Papillary Muscle

In a human papillary muscle (taken from the same heart as the Purkinje fiber of Section 3.1.2), ACh increased APD after 3 min exposure (Figure 1b). 

### 3.2. Optical Mapping Technique

**Figure 2:**
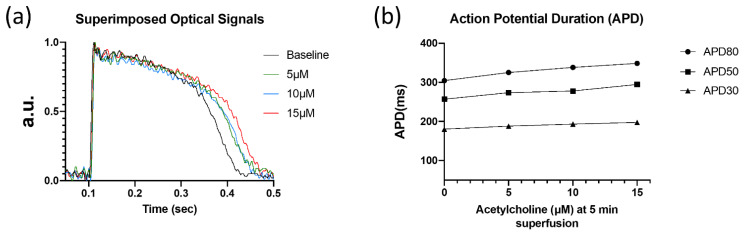
Optical recordings of human left ventricular myocardial slice. (**a**) Superimposed optical mapping recording of human left ventricular APs during control (‘base’) and after superfusion with ACh (5, 10, and 15 µM) at a BCL of 1000 ms. (**b**) AP duration at 30, 50, and 80% repolarization (APD_30_, APD_50_, and APD_80_, respectively) under control conditions and after superfusion with ACh at a BCL of 1000 ms.

In a human myocardial slice preparation, ACh (5, 10, and 15 µM) dose-dependently increased APD (Table 2, Figure 2) at 30, 50, and 80% repolarization (APD_30_, APD_50_, and APD_80_, respectively).

### 3.3. Patch Clamp Experiments

#### 3.3.1. Acetylcholine Decreases I_Kr_ and Shifts Its Activation in hiPSC-CMs

**Figure 3:**
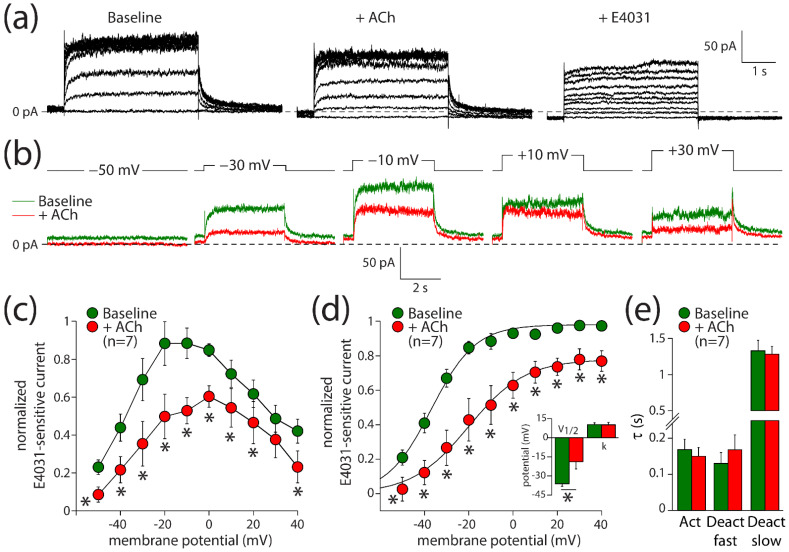
ACh reduces the rapid delayed rectifier K^+^ current (I_Kr_) in hiPSC-CMs. (**a**) Typical current traces in response to 4 s long depolarizing pulses from a holding potential of −50 mV under baseline conditions (left panel) in presence of 5 µM ACh (middle panel) and in additional presence of 5 µM E4031 (right panel). (**b**) Typical E4031-sensitive currents obtained from the currents in panel a under baseline conditions and in the presence of ACh. (**c**) Current–voltage (I–V) relationships of the E4031-sensitive current during the depolarizing steps. (**d**) I–V relationships of the E4031-sensitive current during the tail. Insets: average V_1/2_ (membrane potential for the half-maximal activation) and k (slope factor) of the voltage-dependence of activation. (**e**) Average time constants of activation (Act) and deactivation (Deact). * *p* < 0.05.

Next, we evaluated the effects of ACh on I_Kr_. These experiments were performed in ventricular-like hiPSC-CMs, due to the limited availability of (healthy) human hearts and the rather difficult isolation of human ventricular cardiomyocytes [41,42]. Ventricular-like hiPSC-CMs express muscarinic receptors [43], but they lack I_K,ACh_ [44,45]. I_Kr_ was measured using the perforated patch clamp method to avoid time-dependent changes in delayed rectifier K^+^ current amplitude due to cell dialysis [25]. Figure 3a shows current recordings under baseline conditions (left panel), in the presence of 5 µM ACh (middle panel), and in the additional presence of E4031 to block I_Kr_ completely (right panel). Figure 3b shows the E4031-sensitive current that we defined as I_Kr_ during a series of voltage clamp steps. ACh decreased I_Kr_ significantly over the entire voltage range both during the depolarizing steps (Figure 3c; two-way repeated measures ANOVA; ACh vs. baseline: F(1,6) = 59.710, *p* < 0.001; membrane potential: F(6,9) = 7.430, *p* < 0.001; interaction: F(9,54) = 3.173, *p* = 0.004) and tail (Figure 3d; two-way repeated measures ANOVA; ACh vs. baseline: F(1,6) = 23.303, *p* = 0.003; membrane potential: F(6,9) = 67.967, *p* < 0.001; interaction: F(9,54) = 3.517, *p* = 0.002). For example, the tail current density after depolarizing steps to +40 mV was reduced from 3.6 ± 0.8 pA/pF under baseline conditions to 2.7 ± 0.6 pA/pF in the presence of ACh, which is a decrease of 23 ± 6% (*n* = 7). The V_1/2_ of activation was significantly shifted towards less negative potentials (Figure 3d, inset; paired *t*-test, t(6) = −2.778, *p* = 0.032). Under baseline and ACh conditions, V_1/2_ was −36.1 ± 2.1 and −18.8 ± 5.7 mV (*n* = 7), respectively, indicating a +17.3 mV shift in response to ACh. The slope factor, k, was not affected by ACh (Figure 3d, inset; paired *t*-test, t(6) = 0.322, *p* = 0.758). Neither the time constant of activation nor the time constants of fast and slow deactivation were affected by ACh (Figure 3e; paired *t*-tests; t(6) = 1.347, *p* = 0.227; t(6) = −1.659, *p* = 0.148; t(6) = 0.814, *p* = 0.447; respectively). Thus, ACh decreases I_Kr_ through a positive shift of the voltage of half-maximal activation, in addition to the aforementioned 23% reduction in its fully activated conductance.

#### 3.3.2. I_Kr_ Reduction Contributes to the ACh-Induced Action Potential Prolongation

**Figure 4:**
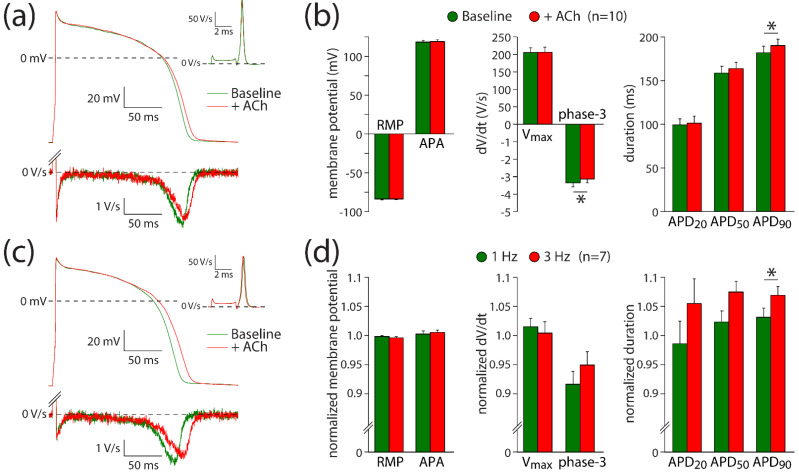
ACh prolongs APs in a frequency-dependent way. (**a**) Typical APs (top panel) and their first derivative of the AP upstroke (inset) and repolarization phases (bottom panel) at 1 Hz under baseline conditions and in presence of 5 µM ACh. (**b**) Average AP parameters at 1 Hz under baseline conditions and in presence of 5 µM ACh. (**c**) Typical APs (top panel) and their first derivative of the AP upstroke (inset) and repolarization phases (bottom panel) at 3 Hz under baseline conditions and in presence of 5 µM ACh. (**d**) Normalized effects of ACh on APs recorded at 1 and 3 Hz. RMP: resting membrane potential; V_max_: maximal AP upstroke rate; APA: AP amplitude; APD_20_, APD_50_, APD_90_: AP duration at 20, 50, and 90% repolarization, respectively; phase-3: maximal phase-3 repolarization rate. * *p* < 0.05.

The ACh-induced effects on I_Kr_ suggest that it may contribute to the ACh-induced AP prolongation observed with the microelectrode and optical measurements. The role of I_Kr_ in AP changes is further explored using hiPSC-CMs under conditions of I_Ks_ blockade with JNJ303. Thus, potential changes of ACh on I_Ks_ [26] will not contribute to changes in AP repolarization. Figure 4a shows typical APs at 1 Hz measured in absence (baseline) and presence of ACh; Figure 4b summarizes the average effects of ACh. ACh prolongs the AP slightly without changes in V_max_, APA, or RMP (Figure 4a,b; paired *t*-tests; t(9) = −0.264, *p* = 0.798; t(9) = −0.876, *p* = 0.404; t(9) = −0.178, *p* = 0.862; respectively). The AP prolongation resulted in a significantly different APD_90_ (paired *t*-test; t(9) = −2.334, *p* = 0.044), while the AP duration in the early phases (APD_20_ and APD_50_) was not affected at a statistically significant level (paired *t*-tests; t(9) = −0.672, *p* = 0.519; t(9) = −1.900, *p* = 0.090; respectively). The APD_90_ increased slightly, but significantly, from 182 ± 8 to 190 ± 7 ms (*n* = 10). Consistent with the ACh-induced effects late during the AP, the maximal rate of phase-3 repolarization was significantly reduced in the presence of ACh (Figure 4a, bottom panel; Figure 4b; paired *t*-test; t(9) = −2.407, *p* = 0.039). The maximal phase-3 repolarization rate decreased from 3.4 ± 0.2 V/s under baseline conditions to 3.1 ± 0.2 V/s (*n* = 10) in the presence of ACh.

Although we observed a small AP prolongation, the net effect of ACh on AP duration is likely a tight interplay between I_Ca,L_ and I_Kr_ changes. At fast pacing rates, the amplitude of I_Ca,L_ decreases, while that of I_Kr_ increases [46,47]. Therefore, we assessed the frequency-dependence of ACh-induced AP changes in seven hiPSC-CMs. Figure 4c shows typical APs at 3 Hz measured in the same cell as used for Figure 4a at 1 Hz. At 3 Hz stimulation, the AP prolongation was more pronounced than at 1 Hz (Figure 4d), with a statistically significant increase in the effect of ACh on APD_90_ (paired *t*-test; t(6) = −2.502, *p* = 0.046), without statistically significant differences in the effects of ACh on RMP, APA, V_max_, phase-3 repolarization, APD_20_, or APD_50_ (paired *t*-tests; t(6) = 1.164, *p* = 0.289; t(6) = −0.557, *p* = 0.598; t(6) = 0.712, *p* = 0.503; t(6) = −1.500, *p* = 0.184; t(6) = −1.906, *p* = 0.105; t(6) = −2.271, *p* = 0.064; respectively). These results indicate that I_Kr_ contributes to ACh-induced AP changes and that its contribution is frequency-dependent. 

### 3.4. Block of I_Kr_ Prolongs Action Potentials

#### 3.4.1. Effect of ACh-Induced Reduction in I_Kr_ on Simulated Human Ventricular Cardiomyocyte

**Figure 5:**
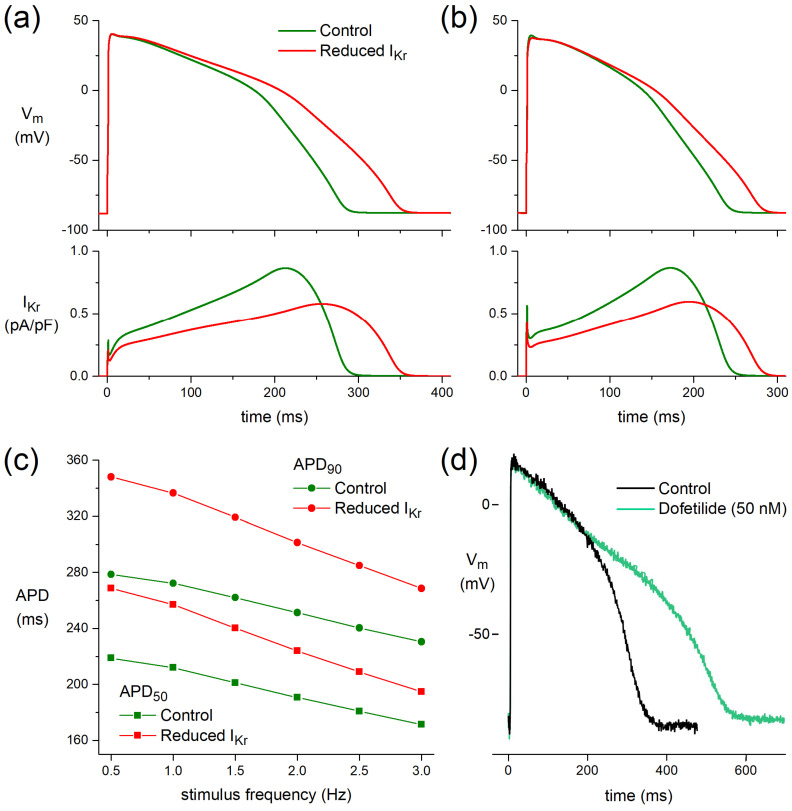
Functional effects of the ACh-induced reduction in I_Kr_ on a model human ventricular cardiomyocyte (O’Hara–Rudy human ventricular cell [27]). (**a**) APs (top) and associated I_Kr_ (bottom) at a stimulus frequency of 1 Hz. (**b**) APs (top) and associated I_Kr_ (bottom) at a stimulus frequency of 3 Hz. (**c**) AP duration at 50% and 90% repolarization (APD_50_ and APD_90_, respectively) at stimulus frequencies ranging from 0.5 to 3.0 Hz. (**d**) Human left ventricular myocardial slice: superimposed recording of APs during control and after superfusion with dofetilide (50 nM) at a BCL of 1000 ms.

At a stimulus frequency of 1 Hz, APD_50_ and APD_90_ of the O’Hara–Rudy model human ventricular cardiomyocyte [27] are increased by 45 and 64 ms, respectively, upon the ACh-induced reduction in I_Kr_ without essential changes in V_max_, APA, or RMP (Figure 5a). These increases in APD_50_ and APD_90_ largely result from a delay in phase-3 repolarization and are smaller at a stimulus frequency of 3 Hz, with values of 23 and 38 ms, respectively (Figure 5b). Figure 5c shows APD_50_ and APD_90_ at stimulus frequencies ranging from 0.5 to 3.0 Hz. 

#### 3.4.2. Dofetilide Prolongs Human Action Potential 

In a human left ventricular myocardial slice preparation taken from a donor heart, selective block of I_Kr_ by 50 nM dofetilide [48] prolonged APD after 60 min exposure (Figure 5d). 

## 4. Discussion

In the current study, we examined the effect of ACh on AP parameters in human ventricular preparations and hiPSC-CMs and the effect of ACh on I_Kr_ in hiPSC-CMs. We found in hiPSC-CMs that ACh decreased I_Kr_ density with a positive shift of the voltage of half-maximal activation. This is consistent with a study of HERG channels—i.e., ion channels composed of the α-subunit of I_Kr_ channels—expressed in CHO cells [49]. Additionally, in the same study the authors found that the IC50 of ACh-induced HERG current blockade was 0.39 ± 0.15 µM. We further established that ACh is capable of prolonging repolarization in human ventricular preparations and hiPSC-CMs and found that the ACh-induced AP prolongation in hiPSC-CMs was more pronounced at high stimulation rate. The latter might have the functional consequence that when the parasympathetic tone is enhanced, which potentially results in lower heart rate, the AP prolongation induced by ACh will be less pronounced. However, in our computer simulations using the O’Hara–Rudy human ventricular cell model [27], we observed the opposite effect, with a less pronounced I_Kr_-induced AP prolongation at higher stimulation rates (Figure 5c). This apparent discrepancy underlines the involvement of membrane currents other than I_Kr_ in the ACh-induced AP prolongation. It might be important to study whether ACh-induced AP prolongation is additive with some drug-induced AP prolongation or proarrhythmic in some conditions, e.g., long QT syndrome (LQTS) or weakened repolarization reserve. Yap and Camm delineated that there was not a simple relation between the degree of drug-induced QT prolongation and the likelihood of the development of TdP [50]. Elevation in parasympathetic tone associated with enhanced release of ACh might further prolong QT and increase the probability of the appearance of TdP in drug-induced QT prolongation. According to our results showing I_Kr_ blocking ability of ACh, increased parasympathetic tone might cause a considerable repolarization lengthening in case of weakened repolarization reserve. Repolarization reserve might be weakened, e.g., in chronic heart failure, diabetes mellitus, hypothyroidism, and hypokalemia (for review see [51]).

The net effect of ACh on AP duration is probably a tight interplay between I_Ca,L_ and I_Kr_ changes. ACh elicited a slight but significant prolongation of APD_90_ in human myocardial slices in our study. This prolongation might be attributable to I_Kr_ inhibition (Figure 3), in combination with changes in I_Ks_ [26], and the prolongation might be limited by I_Ca,L_ inhibitory action of ACh [11,12]. This is supported by our computer simulations (Figure 5a–c), which demonstrate a more pronounced AP prolongation when only the effects of 5 µM ACh on I_Kr_ (Figure 3) were incorporated. The fact that APD_90_ is also increased in our hiPSC-CMs experiment where the cells were pretreated with the I_Ks_ blocker JNJ303 (Figure 4) indicates that the ACh-induced reduction in I_Kr_ is important for the observed AP prolongations. ACh elicited much smaller repolarization lengthening than that of the well-known selective I_Kr_ blockers dofetilide (Figure 5d) and E4031 [52], consistent with the almost complete I_Kr_ block by 50 nM dofetilide and 1 µM E4031 and the only partial inhibitory action of ACh on I_Kr_, which is furthermore counteracted by the simultaneous inhibitory action of ACh on I_Ca,L_.

Gilmour and Zipes demonstrated an ability of ACh to increase APD_50_ in canine Purkinje fibers [21]. We found the same in a human Purkinje fiber taken from a heart with reduced ejection fraction. In addition, we found a close-to-significant increase in APD_50_ in addition to a significant increase in APD_90_ at 3 Hz stimulation in hiPSC-CMs. Therefore, it seems that the effect of ACh on APD_50_ exists in both human and dog. In a meticulous study the presence of M_1_, M_2_, M_3_, and M_5_ receptors in the human heart was demonstrated with different methodologies [53]. Decrease in I_Ca,L_ in heart cells is mediated by M_2_ receptors [54]. I_K_,_ACh_ is not present in healthy canine ventricular cells [10]. It was reported that the stimulation of muscarinic subtype 3 (M_3_) receptors elicited by ACh increased the contractile force, possibly by elevating inositol-1,4,5-trisphosphate (IP_3_) levels in canine cardiac Purkinje fibers [55]. Whether the same is applicable to human cardiac Purkinje fibers is unknown, but it is important to mention that in a study performed on trabeculae obtained from non-diseased human heart ACh (10^−9^ to 10^−4^ M; 1 nM to 100 µM) elicited a positive inotropic effect on the baseline ventricular force of contraction [17].

In the present study, we used ACh concentrations in the micromolar range. There is a clear regional variation of ACh concentrations in the four chambers of the heart [56], and various studies indicate that the normal ACh concentration in the ventricles is 1–2 nM [56,57]. At first glance our used concentrations thus seem supraphysiological, but we cannot exclude that the ACh concentration in vivo is locally higher due to the rich cholinergic innervation of the ventricular subendocardium and subepicardium [58], with ACh levels reaching the millimolar range at the site of release [59]. In addition, the non-neuronal ACh released from cardiomyocytes is supposed to amplify neuronal cholinergic effects (for review, see [58]). Moreover, the used concentrations represent increased parasympathetic tone as may occur upon application of the vagal nerve stimulation protocol on patients in clinical studies rather than the normal concentration. Furthermore, we added ACh to the perfusion solutions, and, as discussed previously, ACh is a positively charged quaternary amine with limited diffusion capability through tissues, which is quickly hydrolyzed by tissue acetylcholinesterase [60,61]. Lin and colleagues [61] even postulate that very high concentrations of ACh are required in tissue studies to simulate normal cardiac conditions. Finally, our used concentrations of ACh were chosen to reflect previous studies and were only slightly outside the range of half-maximal effect doses reported in animal and human ventricular tissue [17,20,60].

## 5. Conclusions

We found evidence for the ability of ACh to prolong APs in human ventricular preparations and hiPSC-CMs. Our results seem to be in accordance with the reports showing that ACh prolonged QT interval and MAP duration in patients [18,19]. The repolarization lengthening observed in the human ventricular preparations and in hiPSC-CMs was at least attributable to I_Kr_ block. Of note, vagal nerve stimulation might be a therapy for heart failure, and it is important to understand the electrophysiological effects of ACh on repolarization in humans.

## Figures and Tables

**Table 1:** Effects of ACh on human myocardial slice action potential parameters at pacing cycle length of 1000 ms.

	RMP	APA	V_max_	APD_90_	APD_75_	APD_50_	APD_25_	APD_10_
	(mV)	(mV)	(V/s)	(ms)	(ms)	(ms)	(ms)	(ms)
Control	−84.5	103.8	115.6	369.5	333.2	284.4	197.7	68.1
(*n* = 3)	±0.8	±4.8	±6.0	±19.6	±9.7	±17.1	±17.4	±31.3
Acetylcholine 5 μM	−83.0	105.1	112.8	371.7	336.9	284.8	187.4	65.4
2 min	±1.4	±5.4	±8.8	±19.4	±10.3 *	±16.3	±14.8	±31.4
Acetylcholine 5 μM	−85.3	104.0	117.7	376.3	340.3	291.9	187.4	66.6
3 min	±0.8	±4.7	±7.4	±18.4 *	±9.5 *	±14.9	±14.5	±32.4
Acetylcholine 5 μM	−85.1	102.9	113.4	378.1	341.7	293.2	188.7	69.8
5 min	±2.5	±3.4	±8.5	±20.8 *	±9.7 *	±14.0	±12.7	±33.2

Data are mean ±SEM. RMP: resting membrane potential, APA: action potential amplitude, APD_90_: action potential duration at 90% repolarization, APD_75_: action potential duration at 75% repolarization, APD_50_: action potential duration at 50% repolarization, APD_25_: action potential duration at 25% repolarization, APD_10_: action potential duration at 10% repolarization, V_max_: maximum rising rate of the action potential upstroke, *n*: number of observations (i.e., number of preparations obtained from different human donor hearts). One-way repeated measures ANOVA; RMP: F(3) = 0.873, *p* = 0.505; APA: F(3) = 0.369, *p* = 0.778; V_max_: F(3) = 0.0821, *p* = 0.967; APD_90_: F(3) = 8.083, *p* = 0.016; APD_75_: F(3) = 13.204, *p* = 0.005; APD_50_: F(3) = 4.765, *p* = 0.051; APD_25_: F(3) = 0.459, *p* = 0.721; APD_10_: F(3) = 1.887, *p* = 0.233. * *p* < 0.05 vs. control.

**Table 2:** Dose-dependence of ACh-induced increase in action potential duration in a human myocardial slice preparation at pacing cycle length of 1000 ms.

	APD_30_	APD_50_	APD_80_
	(ms)	(ms)	(ms)
Control	181	257	305
Acetylcholine 5 μM			
1 min	184	267	315
3 min	180	268	319
5 min	188	274	325
Acetylcholine 10 μM			
1 min	192	277	329
3 min	194	282	331
5 min	192	278	338
Acetylcholine 15 μM			
1 min	196	290	341
3 min	200	294	345
5 min	198	295	349

APD_30_: action potential duration at 30% repolarization, APD_50_: action potential duration at 50% repolarization, APD_80_: action potential duration at 80% repolarization.

## Data Availability

Data will be available after publication upon request to academic researchers.
